# Advanced in developmental organic and inorganic nanomaterial: a review

**DOI:** 10.1080/21655979.2020.1736240

**Published:** 2020-03-06

**Authors:** Khalisanni Khalid, Xuefei Tan, Hayyiratul Fatimah Mohd Zaid, Yang Tao, Chien Lye Chew, Dinh-Toi Chu, Man Kee Lam, Yeek-Chia Ho, Jun Wei Lim, Lai Chin Wei

**Affiliations:** a Malaysian Agricultural Research and Development Institute (MARDI), Serdang, Malaysia; b Department of Chemistry, Faculty of Science, University of Malaya, Kuala Lumpur, Malaysia; c College of Materials and Chemical Engineering, Heilongjiang Institute of Technology, Harbin, PR China; d State Key Laboratory of Urban Water Resource and Environment, School of Environment, Harbin Institute of Technology, Harbin, PR China; e Dalian SEM Bio-Engineering Technology Co., Ltd, Dalian, PR China; f Fundamental and Applied Sciences Department, Centre of Innovative Nanostructures & Nanodevices (COINN), Institute of Autonomous System, Universiti Teknologi PETRONAS, Bandar Seri Iskandar, Malaysia; g College of Food Science and Technology, Nanjing Agricultural University, Nanjing, China; h Sime Darby Plantation Research (Formerly Known as Sime Darby Research), R&D Centre – Carey Island, Pulau Carey, Malaysia; i Faculty of Biology, Hanoi National University of Education, Hanoi, Vietnam; j Centre for Molecular Medicine Norway (NCMM), Nordic EMBL Partnership, University of Oslo and Oslo University Hospital, Norway; k Department of Chemical Engineering, Universiti Teknologi PETRONAS, Seri Iskandar, Malaysia; l Civil and Environmental Engineering Department, Univesiti Teknologi PETRONAS, Seri Iskandar, Malaysia; m Center for Urban Resource Sustainably, Institute of Self-Sustainable Building, Universiti Teknologi PETRONAS, Seri Iskandar, Malaysia; n Department of Fundamental and Applied Sciences, Universiti Teknologi PETRONAS, Seri Iskandar, Malaysia; o Centre for Biofuel and Biochemical Research, Institute of Self-Sustainable Building, Universiti Teknologi PETRONAS, Seri Iskandar, Malaysia Lim; p Nanotechnology & Catalysis Research Centre (NANOCAT), University of Malaya (UM), Kuala Lumpur, Malaysia

**Keywords:** Nanoparticles, nanotechnology, nanopackaging, biotechnology, nanoscience, nanocomposites, nanoparticulates, nanomagnetism, nanoscale

## Abstract

With the unique properties such as high surface area to volume ratio, stability, inertness, ease of functionalization, as well as novel optical, electrical, and magnetic behaviors, nanomaterials have a wide range of applications in various fields with the common types including nanotubes, dendrimers, quantum dots, and fullerenes. With the aim of providing useful insights to help future development of efficient and commercially viable technology for large-scale production, this review focused on the science and applications of inorganic and organic nanomaterials, emphasizing on their synthesis, processing, characterization, and applications on different fields. The applications of nanomaterials on imaging, cell and gene delivery, biosensor, cancer treatment, therapy, and others were discussed in depth. Last but not least, the future prospects and challenges in nanoscience and nanotechnology were also explored.

## 1. Introduction

Nanomaterials is** **defined as a set of substances where at least one of its dimensions is between 1 and 100 nm, nanomaterials follow the central principle of nanoscience and nanotechnology, the application of which covers a wide interdisciplinary of research area and development activity that grows explosively worldwide [1]. Nanomaterials have the ability to transform into functionalized substitute that can be further re-accessed. Common types of nanomaterials include nanotubes, dendrimers, quantum dots (QDs), and fullerenes. The rising awareness of nanomaterials is due to its unique optical properties which are significantly impactful for various fields such as electronics, mechatronics, medicine, pharmaceutical, ionic liquids, polymer, and many more. Commercial applications of nanomaterials include nanoscale titanium dioxide in cosmetics, sunscreen, and self-cleaning windows; nanocoatings and nanocomposites in windows, sports equipment, bicycles, and automobiles; nanoscale silica being used as filler in cosmetics and dental fillings; and others such as stain-resistant and wrinkle-free textiles, electronics, paints, and varnishes [2].

**Figure 1.**
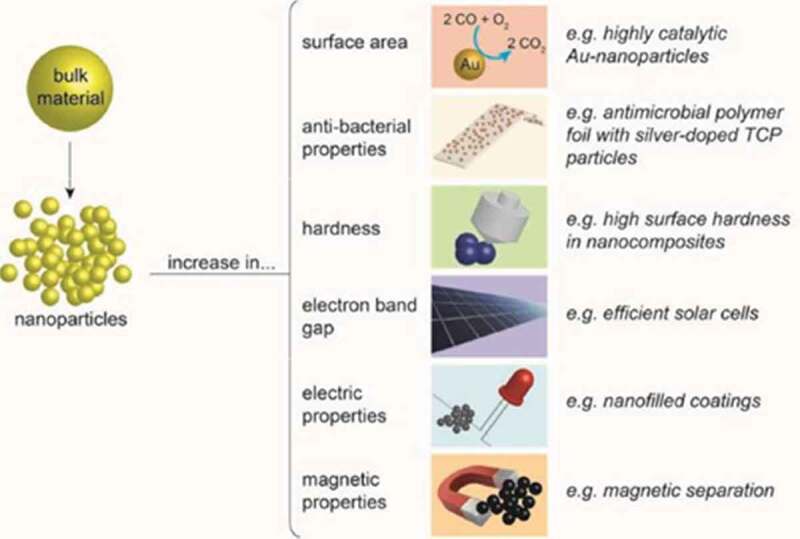
The changes in properties when a bulk material is broken down into nanoparticles (NPs).

Redesigning materials at the molecular level state, also known as engineered nanomaterials, where modification is made in their small size and novel properties, are generally not visualized in their conventional and bulk counterparts. A distinct propery of these nanomaterials is their relatively large surface area which triggers the novel theory of quantum effects. Nanomaterials provide a much greater surface area to volume ratio compared to their conventional forms, which is beneficial as this can provide greater chemical reactivity affected by their specialty [3]. Considering the reaction at the nanoscale level, the material properties and characteristics which lead to novel optical, electrical, and magnetic behaviors can be more vital due to the quantum effects [4]. Nanostructured materials are classified as zero-dimensional (0-D), one-dimensional (1-D), two-dimensional (2-D), and three-dimensional (3-D) nanostructures [5]. These dimensionalities of nanomaterials are characterized using an ultrafine grain size less than 50 nm or limited to 50 nm. Various modulation dimensionalities can be formed such as 0-D (e.g. atomic clusters, filaments, and cluster assemblies), 1-D (e.g. multilayers), 2-D (e.g. ultrafine-grained overlayers or buried layers), and 3-D (e.g. nanophase materials composed of equiaxed nanometer-sized grains). Figure 1 reveals the changes in properties when a bulk material is broken down into nanoparticles (NPs).

The aim of this review is to bring together science and applications on inorganic and organic nanomaterials specifically emphasizing on synthesis and preparation, processing, characterization, and applications of organic and inorganic nanomaterials that provide a novel approach in the field of nanomaterial sciences.

## 2. Evaluation studies of inorganic and organic nanomaterials

Most nanomaterials that are commonly composed of silicon and whose chemical (molecular) properties range from sub-nanometer to few nanometer levels are well-defined crystallographically materials used in supermolecular chemistry[6,7,8,9]. The raw materials used in making glass, quartz, and desiccants consist of particulate properties that define additives and bulk properties. Hardness of materials is associated with properties of ceramics or metals and is rarely allied with polymer. For instance, materials can be value-added into a quality product by adding additive of small and hard particles in conveying hardness [10]. A similar approach applied for UV-radiation absorption, magnetization, color, and reflectivity can be supplementary to a sensitive polymer-based NP. Nanoparticulate additives are already well established, where nanoparticulate carbon was developed early in the twentieth century known as carbon black typically made up of more than 10% of a typical car tire. Obviously, the term ‘nano’ was only coined much later. Likewise, several most established chemical products would be called ‘nano’ which are introduced today. All of these products have been around for decades and are generally safely recognized product. Chemists design molecular products using thousands of well-established building blocks or subunits. Therefore, this will easily identify the suitable modifications to the dye’s functional core part (or the conjugated system), to alter the molecule solubility and material affinity. Last, the idea of chemical modification by incorporating solid magnetization properties on a NP in a product can serve as a coating application on the particle surface.

### 2.1. Organic and inorganic nanoparticles

Organic NPs have been widely investigated, with liposomes, polymersomes, polymer constructs, and micelles being employed for imaging or drug and gene delivery techniques [11]. Meanwhile, inorganic NPs have also attracted researchers’ attention in recent years attributed to their unique material- and size-dependent physicochemical properties, which are incomparable with traditional lipid- or polymer-based NPs. What makes inorganic NPs attractive? It is their physical properties (e.g. optical and magnetic), in addition to their chemical properties such as inertness, stability, and ease of functionalization [12].

### 2.2. Hybrid organic–inorganic nanomaterials

With the possibility of combining interesting properties out of different components, hybrid organic–inorganic nanomaterials have attracted researchers in developing new and smart nanocomposite materials as they demonstrate many novel properties of high interest, such as high catalytic activity, stimulating optical properties, and various physical properties [13]. However, it is still necessary to integrate a polymer matrix or an organic component, which further improves the mechanical, thermal, and chemical properties. This strategy maximizes the interface between the components, shapes the percolation threshold of the NP filler, and optimizes the processability of the integrated polymer-based component [14]. Despite this, the challenge associated in synthesizing these nanomaterials is the incompatible properties that affect the integration of these components.

### 2.3. Metal-containing organic dendrimers

The presence of these organic dendron ligands in metal-containing organic dendrimers has contributed to various applications in drug delivery agents, chemical sensors, and nanoscale catalysts [15]. These organic dendron ligands act as a good stabilizer for metal and semiconductor NPs [16]. Aside from that, these dendron ligands provide a steric crowing effect where closely packed thin ligands toward the shell are formed by long flexible alkyl chain ligands. Steric crowding effect is an ideal approach as this fills up the spherical ligand layer caused by the dendron ligands which are naturally in a cone shape on the surface of the NP. Furthermore, these dendron ligands are also composed of flexible branches, also known as inter- and intramolecular entanglement where slow diffusion process of these small molecules and ions from the bulk solution transfers to the interface between the nanocrystal and the ligand [17].

Till date, Au NPs are one of the most well-explored NP materials because of the simplicity in their synthesis, surface plasmon properties, and amenability to surface functionalization. As for quantum dot (QD) semiconductor NPs, they are beneficial for applications related to biomarkers and to sensing elements due to their interesting quantum size effects and narrow emission bands. This also includes tuning within the whole visible range in electronic transition (e.g. cadmium chalcogenides), and synthesis-related approach can be carried out easily due to their remarkable size control and high crystallinity.

Previous research has reported that the synthesis and characterization of conjugated polythiophene dendrons and dendrimers exhibited broad absorbance and bandgap tunability by varying the generation and connectivity of the macromolecule [18]. These materials exhibited interesting nanostructures on mica and graphite surfaces due to p–p transition state and van der Waals interactions. This included 2-D crystallization and nanowire formation.

### 2.4. Cell biology nanomaterials

Nanoscale range molecules in cell biology, such as proteins, carotenoids, DNA, and others, have already been studied in depth for a long time. It is known that nanomaterials of size <100 nm can easily infuse into the cells, those with the size of <50 nm can enter most cells, while those of <20 nm can transport and permeate along the blood vessels adjacent to the tissue, and leap through the blood–brain barrier. We have reached the stage where NPs can be created and strategized for the use in biomedicine application which is known as ‘theranostic’ approach for diagnostics (imaging) and therapeutics (drug or gene delivery). Their specialty is mainly due to two beneficial factors such as: (1) high surface area to volume ratio, which allows for the attachment of multifunctional components (e.g. fluorescent moiety and targeting molecules) and (2) the possibility of ubiquitous tissue accessibility [19].

## 3. Inorganic nanomaterial

### 3.1. Magnetic nanoparticles (mNPs)

**Figure 2.**
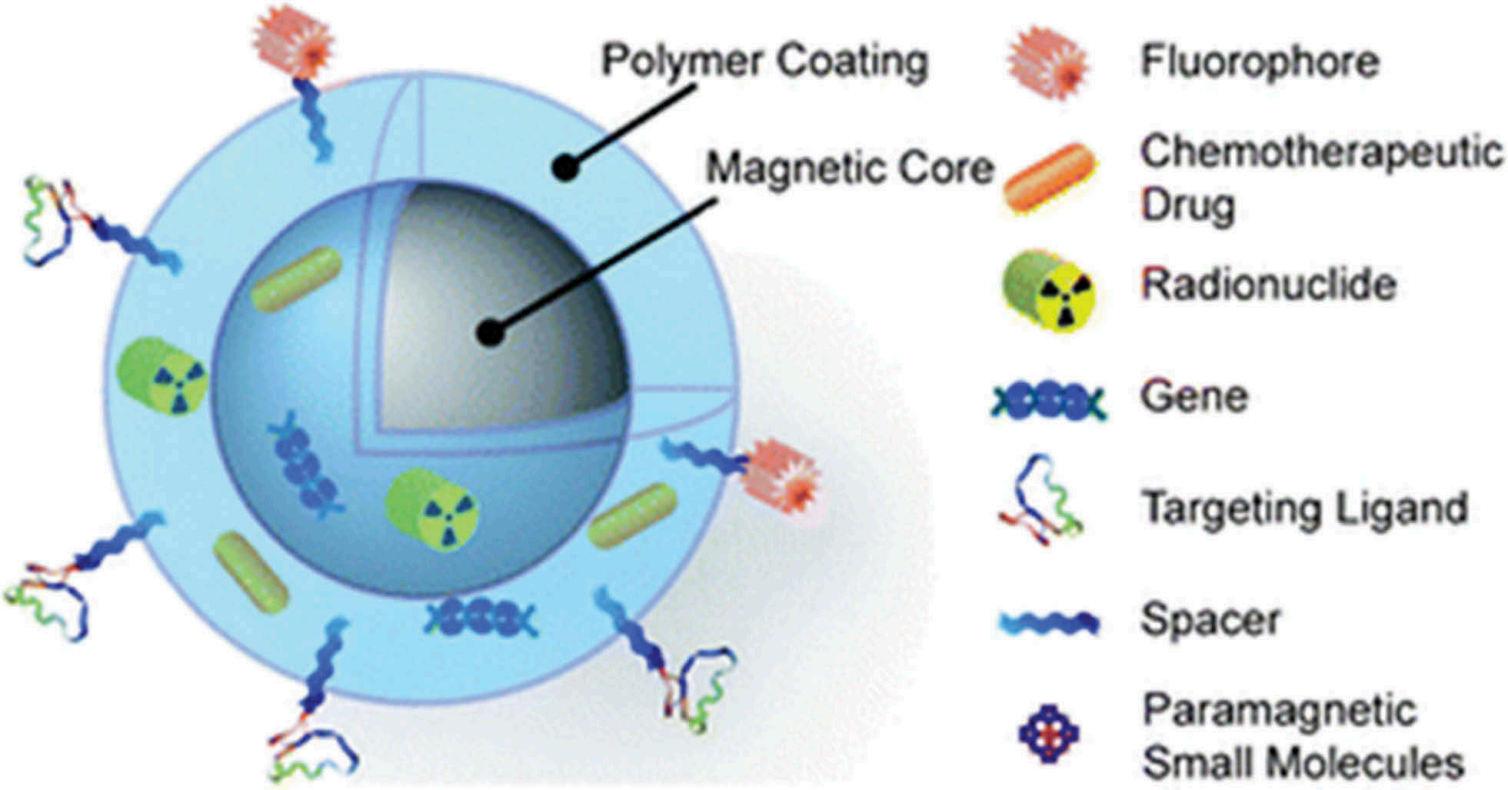
Multifunction magnetic nanoparticles [24].

One of the most profound inorganic nanomaterials is magnetic nanoparticles (mNPs) which are illustrated in Figure 2. They usually consist of a magnetic core (e.g. magnetite (Fe_3_O_4_) or maghemite (g-Fe_2_O_3_)) [20]. Other metals such as cobalt and nickel are also used, but have limited applications due to their toxicity and vulnerability to oxidation [21]. Iron is primarily stored in the protein ferritin in the human body. Iron oxide mNPs have the ability to process excess iron to supply in the human body. The presence of these cationic mNPs remains localized for a long period of time in the endosomes [22]. Then, elemental compounds such as iron and oxygen join the body storage which are being metabolized or consumed by hydrolytic enzymes during the postcellular uptake in the endosome and lysosomes. The iron homeostasis in the human body is well controlled by adsorption, excretion, and storage where the iron level is maintained and postulated. The role of iron oxide NPs helps in processing any excess iron in the body [23]. Iron plays an important role in virtually all living tissues; however, it has limited bioavailability. In some cases, it can be toxic to cells in the form of free iron or as opposed to being associated with hemoglobin.

As for advanced biomedical materials, superparamagnetic iron oxide (SPIO) NPs own a unique superparamagnetic property [25]. When SPIO NPs are being exposed to an external magnetic field, a large magnetic moment will be triggered and subsequently disappeared when the magnetic field is removed. This large moment results in a higher signal change per unit of particles; thus, only smaller quantities are needed to produce good signal feedback [26].

The implementation of biocompatible surface coating to the inorganic iron core provides a stable behavior under physiological conditions (i.e. inhibits aggregation). A variety of substances include synthetic and natural polymer (e.g. proteins or dextran) and amphiphilic molecules such as fatty acids or phospholipids [27] which can be used as coating materials. The controlled surface can be further functionalized by coupling with fragments of DNA strands, proteins, peptides, or antibodies. Findings related to mNPs have been increasingly exploited as efficient delivery vectors, leading to opportunities in magnetic resonance imaging (MRI) contrast enhancement, mediators of hyperthermia cancer treatment, and targeted therapies [28].

#### 3.1.1. Magnetic resonance imaging (MRI)

Due to its noninvasive nature as well as capabilities of providing high 3-D resolution and tomographic, MRI is a powerful imaging tool and offers the advantage of high spatial resolution of contrast differences between tissues. Compared to the conventional gadolinium chelates, the mNP-based contrast agents offer excellent image enhancement due to their large magnetic moment as well as improved cellular internalization and slower clearance from the target site [29]. U.S. Food and Drug Administration has approved the use of iron oxide mNPs for use as MRI signal enhancers. For MRI purposes, iron oxide cores are commonly used as so-called T2 contrast agents, due to their ability to shorten T2 relaxation times. These agents can be divided into either SPIOs, with diameters of more than 50 nm, or ultrasmall SPIOs (USPIOs), with diameters of less than 50 nm, which then tend to have longer plasma half-lives of 14–30 h [30,31]. Currently, there are several formulations for clinical applications such as bowel (Lumiren and Gastromark) and liver or spleen imaging (endoderm and feridex). As with all NPs, the tissue distribution is heavily influenced by size; therefore, larger SPIOs tend to rely on passive targeting, such as uptake by the cells of the reticuloendothelial systems (RES), rather than direct labeling, and the USPIOs benefit from slower opsonization and RES clearance.

The next generation of active targeting contrast agents is current ongoing research, which provides exciting new opportunities for imaging, diagnosis, and treatment. The first targeting agents were monoclonal antibodies that exploit molecular recognition to deliver mNPs [28]. However, one of the drawbacks of using monoclonal antibodies is their large size, which can cause poor diffusion through typical biological barriers. MRI agents using mNPs have been evaluated intensively to improve the diagnosis of solid tumors. This is an active area of research, since clinicians require a contrast agent that specifically targeted malignant tumors to allow a more accurate and precise diagnosis of stages of the disease. Direct tumor targeting using antibodies has been successful for rectal carcinoma and breast cancer, as despite the size issue, antibodies remain beneficial in their high specificity. A recent application utilizing antibody-directed targeting is more efficient as the presence of shorter and smaller single-chain fragments (≤20%) provides a high binding affinity and specificity. Antibody-directed targeting consists of antibody heavy- and light-chain variable domains connected by a flexible peptide linker [32]. Conventional iron oxides, however, still provide a poor signal enhancement compared to that with other imaging modalities (e.g. fluorescence and positron emission tomography). Further research is required for developing high and tunable nanomagnetism iron oxide NP [33,34].

#### 3.1.2. Hyperthermia

The growth of tumor cells is more in temperature-sensitive cells compared to other normal healthy cells. Intracellular hyperthermia is a method developed using mNPs (in particular SPIOs) that have been potentially utilized to treat cancers [35]. This treatment consists of a delivery agent which acts as a nanoscale heater function to heat up the cell and instigate necrosis [36,37]. When the nanoscale heater was subjected to an alternating magnetic field, it converts the electromagnetic energy into heat energy, which can be dissipated to the surrounding medium. Magnetic materials with temperature range within 42°C to 60°C provide an effective treatment as these materials can potentially substitute as an in-vivo temperature control switch to prevent overheating of the neighboring healthy tissue [38].

Tumors can be targeted passively via general biodistribution, as tumor tissue tends to have ‘leaky’ vasculature, which allows NPs to accumulate. Alternatively, solid tumors can be directly injected with mNPs and further exposure to an alternating magnetic field for inducing tumor regression. It has been reported that the mNPs can be heated effectively (10–30 nm iron oxide particles) in human cancer models (e.g. breast cancer) [39]. However, the optimization in controlling the heat distribution is needed to be explored to deliver the best performance of it. For example, a hyperthermia research uses tumor-targeted mNPs to evaluate their enhancement of drug payload [40]. The likelihood of targeting chimeric L6 monoclonal antibody has target-specific breast cancer using dextran-coated mNPs. On the other hand, hyperthermia research has also involved targeting gene expression in tumor [41]. It is essentially important to control and evaluate these gene expressions in tumor [42-47] .

#### 3.1.3. Magnetic transfection

The term ‘magnetic transfection’ is used when nucleic acid delivery is being influenced by a magnetic field acting on nucleic acid vectors that are conjugated to mNPs. Magnetic transfection technique can also be applicable for both ‘large’ nucleic acids and small constructs [48]. These mNPs aim to bind these negatively charged DNAs to the mNPs through electrostatic interactions and subsequent release of them after cell internalization [49]. Magnetic transfection has significant advantages over conventional transfection methods such as reduced process time and the increased efficiency in the transfection rate with lower vector doses. Due to the limited half-life in-vivo, these therapies still lack specificity instigated by the poor diffusion process across the cell membrane, resulting in poor magnetic transfection efficacies. Therefore, it is recommended that these mNPs should be used as carriers to overcome some of these problems.

RNA interference is a natural cellular process used to silence gene expression, which can be exploited artificially [50]. The use of wide-scale therapeutic RNA silencing requires the development of a suitable transfection method that can administer siRNA molecules into human cells in vivo. NPs are attractive delivery vehicles for siRNA due to their multifunctionality, allowing them to overcome many problems associated with systemic siRNA application for human cell transfection. A study has shown that NPs can prevent rapid excretion of siRNA by the kidney [51]. This is a fate that succumbs naked siRNA molecules. Magnetic force pulls mNPs toward target cell attachment to NPs and results in minimal renal filtration, extending the unmodified siRNA half-life within the circulation [52]. In 2007, a study demonstrated cancer cell transfection by NP-delivered siRNA, reporting the uptake of mNPs into tumors [53]. In this case, membrane translocation was facilitated by an attached myristoyl-coupled polyarginine peptide. In addition to delivering siRNA treatment, these NPs were further functionalized with a near-infrared dye to allow noninvasive imaging of their localization within the body. The imaging was performed by both MRI and near-infrared in vivo optical imaging. Imaging results from this study showed NPe accumulation in tumors, which is most likely passive due to the enhanced permeability and retention effect associated with the leaky vasculature of tumors. The study concluded that NPs further functionalized with tumor-targeting moieties would increase tumor localization in order to deliver siRNA treatment.

### 3.2. Gold and silver nanoparticles

Colloidal gold or gold nanoparticles (AuNPs) can be easily synthesized for the use of various applications due to its versatility as indicator and detection probe. While AuNPs share most of their attractive qualities with regard to bioapplications with other NPs (e.g. size, inertness, ease of synthesis, and biocompatibility), AuNPs are very attractive candidates for biological imaging techniques as they can be visualized based on the interaction between the NPs and light, whereby the particles strongly absorb and scatter visible light [54]. Upon light absorption, the light energy excites the free electrons in the gold particles to a collective oscillation, the so-called surface plasmon. This absorption lies in the visible region for gold, silver, and copper, with the surface plasmon resonance (SPR) of AuNPs being visible down to 3 nm. AuNPs give rise to both absorption and scattering, the size of which depends on AuNP size [55]. Particles smaller than 20 nm essentially show absorption, but larger sizes of 80 nm increase the ratio of scattering to absorption. On the other hand, silver nanoparticles (AgNPs) have excellent antimicrobial activity against viruses, bacteria, and other eukaryotic microorganisms. Therefore, AgNPs have wide applications in dental materials, textile fabrics, coating stainless steel materials, water treatment, medicine for burn treatment, and others.

#### 3.2.1. Biological imaging

AuNPs have been employed as a contrast agent in electron microscopy for several decades. Owing to the high atomic number of gold, the colloidal gold particles are electron dense which render them among the best for electron microscopy. However, AuNPs were involved in several other imaging techniques that rely on the plasmon band [56]. AuNPs greater than 20 nm can be used in optical microscopy under phase contrast or differential interference contrast mode. Other techniques include fluorescence microscopy, photothermal coherence tomography (similar to ultrasound with good depth penetration), multiphoton SPR microscopy, and X-ray scattering [57].

Perhaps, the most referenced application of AuNPs in bioimaging is in immunostaining. Essentially, antibody-conjugated AuNPs are designed to bind against antigens on fixed and permeabilized cells, being subsequently visualized via TEM or light microscopy [56]. Typically, an excess of gold is used so that virtually all entities are labeled to improve contrast. As cells are fixed and permeabilized, targets outside as well as inside the cells can be labeled. As an ultrastructural marker for detection of proteins, peptides, or amino acids, gold can be used for immunostaining thick or thin sections prior to embedding, or for immunostaining ultrathin sections after embedding [58]. By virtue of its particulate nature, gold as an immunolabel facilitates a semiquantitative analysis of antigen densities on ultrathin sections. Various combinations of different-size gold particles, or dual immunolabelling with enzymatic immunolabels, together with colloidal gold or silver-intensified gold, serve well for ultrastructural immunocytochemical localization of two antigens in the same tissue section [59]. Compared to fluorescence microscopy, AuNPs are more stable as they do not suffer from photobleaching, and in most cases of TEM imaging, AuNPs offer excellent lateral resolution with high contrast.

#### 3.2.2. Cell delivery vehicles

**Figure 3.**
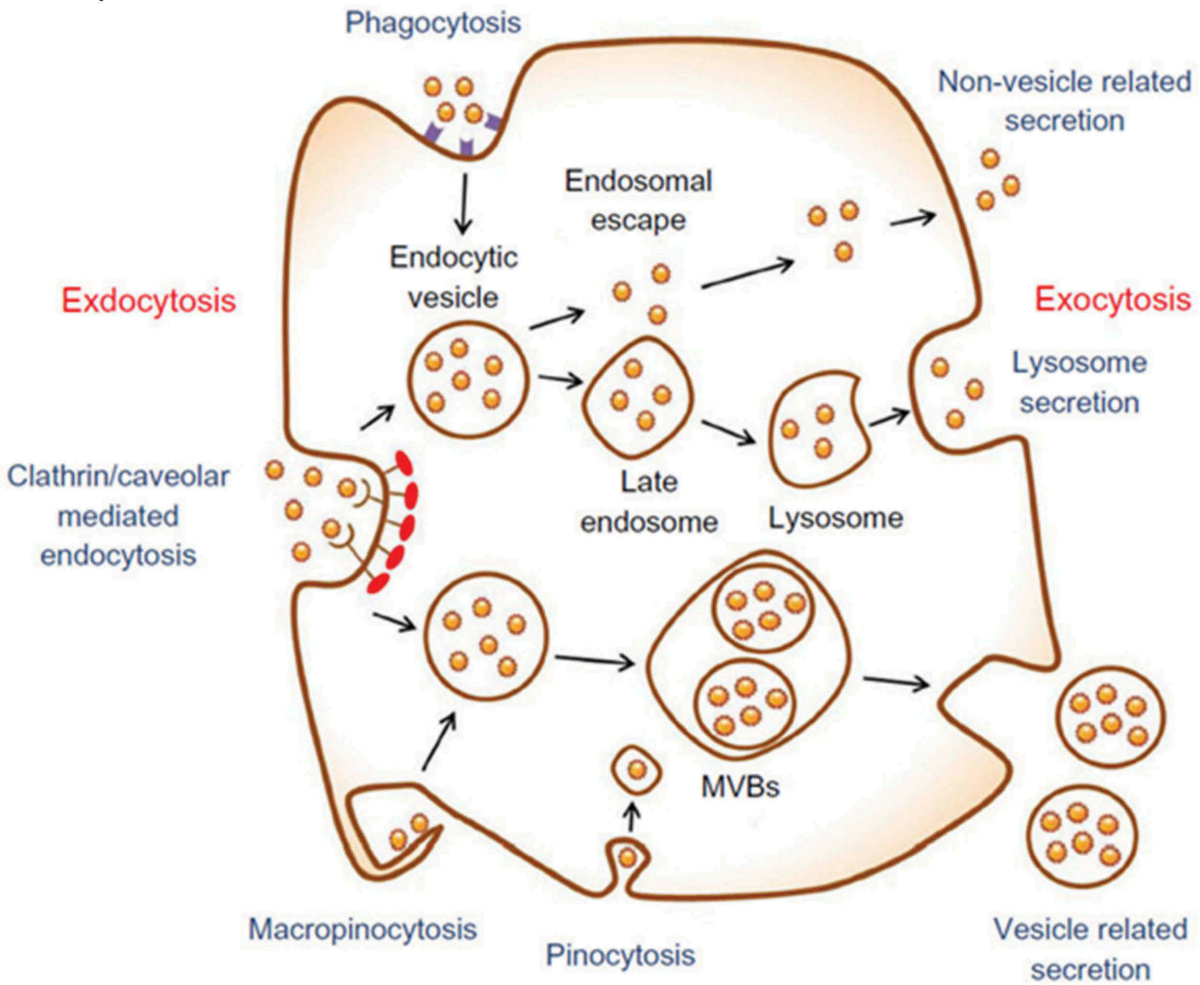
Nanoparticles (NPs) stored inside cells [61].

Most delivery strategies, such as using cancer-targeting moieties conjugated to NPs for delivery into cancer tissue, are very similar to those used for magnetic and other types of NP. Indeed, gold has been used for many years to deliver molecules into cells. For delivery applications, AuNPs are employed for their small size, colloidal stability, ease of synthesis and conjugation, and their inert, biocompatible nature. Introduction into cells can either be forced, for example, with gene guns, or be achieved via cellular uptake. With regard to the gene gun technique, DNA is adsorbed onto the surface of AuNPs, which are then essentially shot into cells [60]. The force required typically provided via gas pressure or electric discharge. While gene guns were more commonly employed in plant cell biology to breach the plant cell wall. Gene guns have also been used for DNA delivery into animal cells. Alternatively, specific or nonspecific endocytosis cellular uptake can be relied upon for AuNP delivery. Specific uptake depends on receptor–ligand binding, for example, using transferrin adsorbed onto the AuNPs as a means of instigating cellular uptake, and is far more effective than passive nonspecific uptake. Following uptake, Figure 3 illustrates the NPs which are stored in endosomal/lysosomal vesicles inside the cells.

There have been efforts to avoid endosomal uptake and subsequent vesicle storage. The nuclear translocation of AuNPs (10–35 nm) coated with nucleoplasmin (a Xenopus oocyte protein that contains a well characterized nuclear localization sequence) was performed using microinjection on chemically modified cells that bypassing the plasma membrane entry [62]. Further work showed nuclear targeting was only achieved when exposing cells in culture to AuNPs (20 nm) derivatized with bovine serum albumin and functionalized with a variety of short peptide sequences that exhibit nuclear localization sequences, when the whole peptide was present, thus including the sequence for plasma membrane translocation (otherwise the particles were trapped in the endosome). Following the advent of cell-penetrating peptide research, whereby direct plasma membrane translocation was achieved, conjugating t-t peptide to AuNPs also achieved excellent cellular uptake levels [63].

#### 3.2.3. Biosensor

While imaging and delivery shown above tend to use passive methods, plasmon-related sensing uses AuNPs in a more active role. Basically, the NPs are required to specifically register the presence of analyte molecules and provide a concentration readout. This is typically achieved by changes in the optical properties of the AuNPs. The plasmon resonance frequency is a very reliable intrinsic feature of AuNPs that can be used for sensing [64]. The binding of molecules to the particle surface can change the plasmon resonance frequency directly, which is visible by scattered light. In addition, the influence of the inter-AuNP distance on the plasmon resonance, when this distance is reduced to less than the particle diameter, is the crucial factor in the sensor application, and linking the NPs with a biological analyte results in a color change that makes the basis of sensing [65]. The first colorimetric sensing of nucleic acids was reported and is now the most recognized example of a gold-based biosensor [66]. The DNA linked the AuNPs together with an interparticle distance of 0.34 nm, which caused a red-to-purple color change depending on whether the gold particles are free in colloidal suspension (red) or attached to the DNA target (blue-violet) [56]. The color change is temperature reversible. Therefore, when the sample is heated, even a single sequence mismatch will result in a different melting temperature, thereby causing a difference in color change. AuNPs possess excellent fluorescent properties and display antiphotobleaching behavior under strong light illumination. AuNPs exhibit strong native fluorescence under relatively high excitation power. For example, if cells stained with AuNPs are illuminated with strong light, AuNP fluorescence can be recorded for cell imaging. A further phenomenon is that the fluorescence of many fluorophores is quenched when they are in close proximity to gold, and this effect can be used for several sensing strategies [67].

### 3.3. Quantum dots

QDs are colloidal nanometer-sized crystals, comprising atoms of elements from groups II to VI (e.g. Cd, Zn, Se, Te) or III to V (e.g. In, P, As) in the periodic table [68]. In terms of bioapplications, the most typical QDs employed were composed of CdSe and encased in a ZnS shell in order to protect from the highly toxic cadmium. By confining the electrons in variable sizes, the energy bandgap of the absorption spectra allows their corresponding emission wavelengths to be tuned from the ultraviolet to near-infrared (NIR) region [69]. Smaller QDs demonstrate blue fluorescence emission (within 380–440 nm), while larger particles demonstrate red fluorescence emission (within 605–630 nm). QDs have further advantages over organic dye molecules, aside from their tuneable fluorescence. They are robust and stable light emitters due to their inorganic makeup and are less susceptible to photobleaching than organic dye molecules. This photostability makes them extremely useful in observing cells over longer periods of time.

#### 3.3.1. Biological imaging

Due to their high photostability and limited cytotoxicity, QDs appeared as a very promising probe for longer-term experiments in living cells, provided several issues are addressed. Initially, water solubility is an important requirement for in vitro and in vivo imaging. Thiol groups (SH) are generally anchored to the ZnS shell with terminal carboxyl (COOH) in order to increase the hydrophilicity of QDs [70] followed by cell internalization. As with both magnetic and gold NPs, the cell uptake is typically enhanced via particle surface functionalization. Several methods have been proposed to deliver QDs to the cell cytoplasm. Aside from the physical methods, such as microinjection and electroporation, many attempts have been made using lipid- or polymer-mediated endocytosis and peptide-mediated endocytosis [71]. The main issue here is that the QDs remain trapped in the endosomes for several days. An alternative pathway is to exploit pinocytosis. This is activated by an increase in osmotic pressure in the cell culture medium. To counter-balance this, the cell uptake medium in the pinosomes is placed together with QDs in the medium. Pinosomes can subsequently be disrupted by a second osmotic shock, thereby releasing the QDs into the cell cytoplasm [72].

#### 3.3.2. Single-cell imaging

Tracking of single cells was initially developed to study membrane receptor dynamics. The first studies included diffusion of transmembrane proteins using micrometer-sized beads of AuNPs [73]. Recently, similar experiments have been performed using QDs to target membrane proteins and study the mobility and kinetics of receptors, transmembrane proteins, and synapses [74]. QDs can be detected and tracked with the same approach as for traditional organic dyes. In the cellular context, tracking QDs is technically difficult. First, the background noise due to cell autofluorescence and to the surrounding QDs diminishes precision. To reduce this noise, QDs emitting in the red field are used, where the cell autofluorescence disappears [75]. Furthermore, using small levels of QDs helps to improve the signal-to-noise ratio. The second difficulty arises from the need to track in a 3-D space. In recent years, various techniques have been developed to acquire image stacks by reconstructing the 3-D structure of the cell. For example, the total internal reflection fluorescence microscopy, where only particles closest to the glass coverslip are excited typically 100–200 nm of a cell [76]. The most popular reconstruction of a z-series, however, is the scanning confocal microscope, which limits excitation to a particular volume.

#### 3.3.3. In vivo imaging

**Figure 4.**
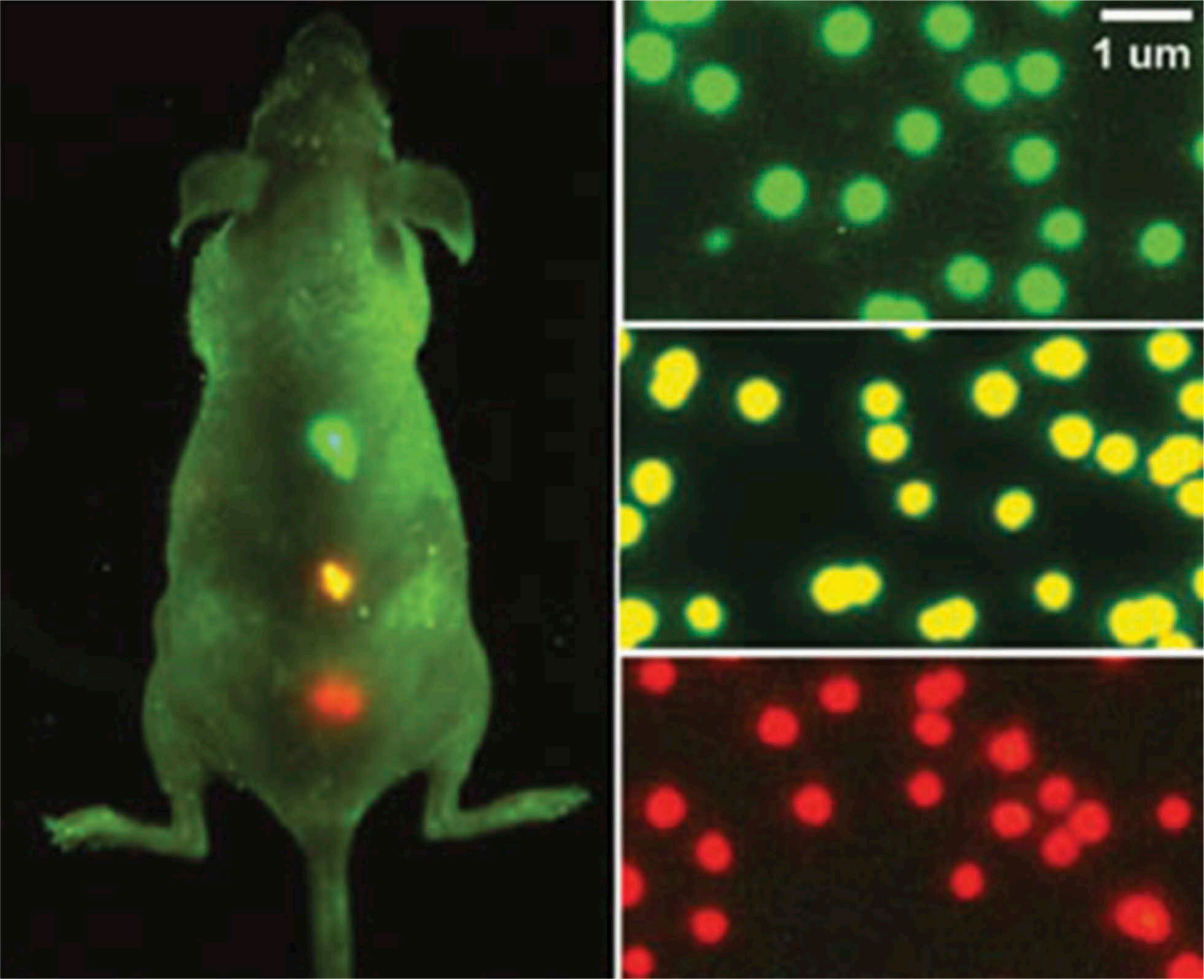
The QDs emitting different wavelengths [79].

QD–peptide conjugates were the first used in vivo to target tumor vasculature in mice shown in Figure 4. Histology indicated that the tissue-specific peptide coating on (CdSe) Frontiers of Nanoscience ZnS QDs increased NP accumulation at vascular sites following intravascular injection [77]. Although this does not describe QD imaging in a live animal, this study demonstrated the potential of using QDs for molecular-level detection. This indicated that untargeted or passive targeting of QD probes demonstrated weak or no signal, but antibody-conjugated QDs resulted in intense fluorescent signals. Despite using targeting moieties with QDs, such as antibodies or peptides, targeted to tumors in live animals for cancer imaging, light penetration and autofluorescence of deep tissue remain a major hurdle. As with single-cell imaging above, the use of red field emitting QDs has minimized light absorption by blood and water and improved tissue depth [78].

#### 3.3.4. Targeted therapies

Similar to mNPs employed in transfection therapies in vivo, QDs are also used as delivery and reporter systems. A big advantage of NP transfection, compared to other types of delivery vehicles, is that they can be functionalized with many different oligonucleotides and cell-binding ligands at once, potentially allowing multiple gene knockdowns and higher affinity for the target cell simultaneously. Studies reported that one siRNA per particle in conjunction with >15 peptides, or two siRNA per particle in conjunction with <10 peptides, gave optimal knockdown and targeting [80].

Further study showed that by using QDs designed with tumor targeting, facilitated by the attachment of a tumor-homing peptide (F3), which binds to nucleolin expressed on the surface of cancer cells [81]. The addition of F3 increased the QD tumor cell uptake by two orders of magnitude compared to nontargeting QDs. Diagnostic imaging was facilitated via the QD core with emission in the NIR, and treatment delivery was facilitated by the attachment of siRNA. This siRNA silenced the enhanced green fluorescent protein gene, but the authors noted that their design could also be applied to silence oncogenes for cancer treatment. Results showed that siRNA attached to the QD by a disulfide bond increased gene knockdown compared to siRNA attached to the QD by a covalent bond. This research demonstrated the importance of such acute attention to molecular design in the development of an efficient NP-based RNAi platform.

### 3.4. Carbon nanotubes

Hollow and porous NPs, such as nanotubes, nanoshells, and hollow spheres, can be loaded with a large amount of cargo, thus enhancing signal and sensitivity. Carbon nanotubes are cylindrical graphene sheets. Although most applications of carbon nanotubes have focused on microelectronic devices, due to their unique electronic and physical properties, carbon nanotubes have shown some attractive properties for biomedical use, including easy translocation across the cell membrane and relatively low toxicity [82]. Both single-walled carbon nanotubes (SWCNTs; 1–3 nm in diameter and 5–30 nm in length) and multiwalled CNTs (MWCNTs; 10–150 nm in diameter and 200 nm to several microns in length) have been investigated for various bioapplications. As with QDs, it has been indicated that SWCNTs have strong optical absorbance in the NIR region where biological systems are known to be highly transparent [83].

#### 3.4.1. Neuronal tissue engineering

CNTs have been rapidly developing as a technology platform for designing novel neuroimplantable devices. They combine incredible strength with extreme flexibility, in addition to exhibiting physical and chemical properties that allow them to efficiently conduit electrical current in electrochemical interfaces. Thus, CNTs can be employed in tissue engineering scaffolds, organized as fibers or tubes, with diameters similar to those in neuronal processes such as axons and dendrites [84]. The first application of nanotubes to neuroscience research employed MWCNTs for growth of rat brain neurons [85]. It was noted that on unmodified tubes neurons extended only one or two neurites, which exhibited very few branches. In contrast, neurons grown on tubes coated with a bioactive molecule (4-hydroxynonenal) demonstrated multiple neurites with extensive branching.

#### 3.4.2. Imaging and cancer treatment

Similar to mNPs and AuNPs, SWCNTs have been utilized in thermal necrosis of cancer cells. Intratumoural injection of tubes, alongside NIR irradiation, resulted in thermal death of human epidermoid mouth carcinoma KB tumor cells in xenografted mice with minimal side effects up to 6 months after treatment, with excretion via urine in 3 months. Similarly, thermal cancer therapy was applied to mice bearing kidney tumors using MWCNTs, and complete tumor regression was observed with no recurrence within 3 months. Tumor targeting has also been employed, using antibodies and folate receptors (which are known to be highly expressed in a range of tumor cells).

When considering targeting, CNTs have also been studied for their use in delivering therapeutic drugs to tumors. SWCNTs exhibiting paclitaxel conjugated to PEG chains were injected into breast tumor xenografts in mice. Particles were well dispersed, with inhibition of tumor growth by almost 60%. Similar studies using other drugs, such as doxorubicin-loaded SWCNTs, RGD peptides to target-specific integrins, and anticancer agent cisplatin, all had varying effects on tumor growth and some retention in the liver, kidney, and spleen [86]. There are other types of inorganic NPs, such as silica, nanoshells, nanorods, and hybrid particles, but the particle systems described above are the four most commonly used when considering bioapplications. Such systems, due to their multifunctional approach, hold a great promise in diagnostics, drug and gene delivery, sensing and biosensing, and both in vitro and in vivo imaging.

While each of the particles described exhibits some features that are original to them, the bioapplications do overlap with many sharing functionalities and targeting groups. Each particle type is designed with the view to boosting cellular uptake efficiency, for image/signal enhancement or cargo delivery and to target-specific tissues/cells [87]. It is envisaged that diseases may be managed by multifunctional NPs that encompass both imaging and therapeutic capabilities, thus allowing simultaneous disease monitoring and treatment.

## 4. Organic nanoparticles

### 4.1. Use of nanoparticles in nucleic acid delivery

Safe and effective gene delivery systems are tremendously important in gene therapy for tackling various genetic diseases, viral infections, and cardiovascular disorders. Gene therapy is a process by which genetic material in the form of oligonucleotides or plasmids is introduced into specific target cells to recover or induce the expression of a normal protein to treat human disorders. Gene therapy can also deliver antisense oligonucleotides or small interfering RNA to interrupt the function of target genes and trigger silencing [88]. In recent decades, many methods for gene delivery have been developed for a wide range of cells and tissues. In addition to these therapeutic applications, gene delivery is widely used in vitro as a research tool to investigate gene function/regulation within a cellular and physiological context. Initial systems for gene delivery have been developed, with the potential to significantly impact biotechnology, diagnostic applications, and basic research.

Gene delivery systems should enable the formation of stable complexes with nucleic acids, providing a low cytotoxicity, and disassemble intracellularly to release the nucleic acid [89]. Environmental interactions are manipulated through the incorporation of functional groups that may stabilize the vector in the extracellular milieu, target a specific tissue or cell type, or maintain the vector within the delivery location. Intracellular trafficking addresses the need to deliver the plasmid to the nucleus for expression; thus, bioactive groups can be incorporated to facilitate endosomal escape or nuclear localization.

Gene delivery systems include viral and nonviral vectors. Viral vectors are the most effective, but there is an overlapping concern about their application, which is limited by their DNA loading capactiy, and in therapeutic application by their oncogenicity and immunogenicity [90]. Nonviral vectors are more reproducible, do not present DNA size limits, and are safer and less costly than viral vectors. Nonviral transfection systems are usually composed of cationic peptides, cationic polymers, or cationic lipids, although the combination of some of them is also possible. These so-called modular nonviral vectors consist of a vector backbone modified with functional groups that mediate environmental interactions and intracellular trafficking to overcome the multiple barriers to gene transfer. For both in vitro and in vivo applications, nonviral vectors must be designed by taking into consideration their interactions with serum components, the extracellular milieu within the tissue or cell culture media, and binding to the cell surface. Many of the aforementioned vectors interact with serum proteins that can inactivate the complex or promote clearance from the desired tissue, which limits the opportunity for cellular internalization. Cellular internalization can be improved by the addition of receptor ligands to the NP, thus increasing binding to cell-surface receptors and allowing the targeting of specific cell types.

Once the DNA is introduced into the cell, it must pass a series of barriers that could damage it, before reaching the nucleus. To avoid this, extensive research is underway to define the ideal carrier to facilitate cell entry and transport to the nucleus of the DNA in a protected manner. The carrier should be sufficiently small to allow cellular internalization and pass through nuclear pores, must have flexible tropism, and should be able to escape the endosome–lysosome process that follows endocytosis [91]. It must also not be cytotoxic nor elicit an immune response. All these conditions can be accomplished by nonviral nanovectors including cationic molecules such as cationic lipids and synthetic or natural cationic polymers, which have been widely developed to condense DNA and to efficiently deliver therapeutic genes within mammalian cells.

While gene delivery has been tested in many cell types using a number of different conditions and readout systems to test for transfection efficiency and toxicity, the lack of a reference standard makes it difficult to directly compare different approaches. Thus, this section presents selective approaches with illustrative examples, rather than an exhaustive comparison of the different methods that have been developed over the years.

### 4.2. Engineered biomaterials for vector backbones

Numerous cationic lipids and polymers have been developed to package DNA for cellular internalization and protect it from degradation, leading to the identification of some structure–function design relationships. Cationic lipids are composed of three basic constituents: a polar headgroup, a linker, and a hydrophobic moiety. The cationic headgroup promotes the interaction with DNA, whereas the hydrophobic moiety provides self-association to form either micelles or liposomes in the presence of a helper lipid, such as dioleylphosphatidylethanolamine. Lipoplexes form a multilayered structure consisting of the plasmid sandwiched between the cationic lipids [92]. Despite the fact that cationic lipids have low immunogenicity, they show in vitro and in vivo cytotoxicity and relatively low gene transfer efficiency [93].

#### 4.2.1. Cationic polymers

Cationic polymers can be classified into two groups, namely natural polymers (e.g. proteins and peptides) and synthetic polymers (e.g. polyethylenimine, dendrimers, and polyphosphoesters). They spontaneously associate with plasmids by electrostatic interactions due to protonatable amine residues to form condensed complexes called polyplexes. With these electrostatic forces between the polymer and the nucleic acid, the complex should maintain a stable and condensed nanosize structure, promoting cellular endocytosis, and possibly enhance the transfection efficiency of therapeutic genes. Furthermore, DNA plasmids can be condensed inside the NPs for protection against nuclease degradation [94]. High-molecular-weight polymers tend to form relatively stable, small complexes compared to low-molecular-weight polymers. Low-molecular-weight polymers, however, can enhance transfection efficiencies, likely due to a decreased cytotoxicity and the increased ability of the plasmid to dissociate from the cationic polymer. Block copolymers can potentially regulate the assembly and structure of the complex, while providing for multiple functions due to each component. Poly(2-diethylaminoethyl methacrylate) or poly(2-dimethylaminoethyl methacrylate), which have primary and secondary amines to facilitate complexation and intracellular trafficking, can be combined with poly(ethylene oxide) (PEO) or poly(propylene oxide) to prevent aggregation and reduce toxicity [95]. Although the condensing vectors seem to be an excellent substitute for viral vectors, some drawbacks inherent in the condensing system limit their application for systemic delivery. These include toxicity of the cationic polymer or lipid, rapid clearance by the reticuloendothelial system, inability of the complex to escape from the endosome/lysosome compartments in the cells, and lack of intracellular unpacking of the nucleic acid construct from the electrostatic complex.

Noncondensing lipids and polymers possess either a neutral or net negative charge. They can be used to engineer nanoscale vectors for tissue- and cell-specific delivery and allow for enhanced transfection efficiency with significantly less toxicity concerns. Nucleic acids are encapsulated within such vectors either by physical entrapment within the matrix or through hydrogen bonds between polymer and nucleic acid bases [96]. Physical encapsulation offers protection from the enzymes and other plasma proteins during its transit from blood to the site of action. Cellular uptake is facilitated since masking the negative charge of DNA prevents electrostatic repulsion with the negatively charged cell surface. Moreover, in contrast to condensing lipids and polymers, the absence of positive charges on noncondensing systems limits their recognition by the mononuclear phagocyte system and hence limits their early clearance and opsonization by IgM and the innate immune response [97].

#### 4.2.2. Solid lipid nanoparticles

**Figure 5.**
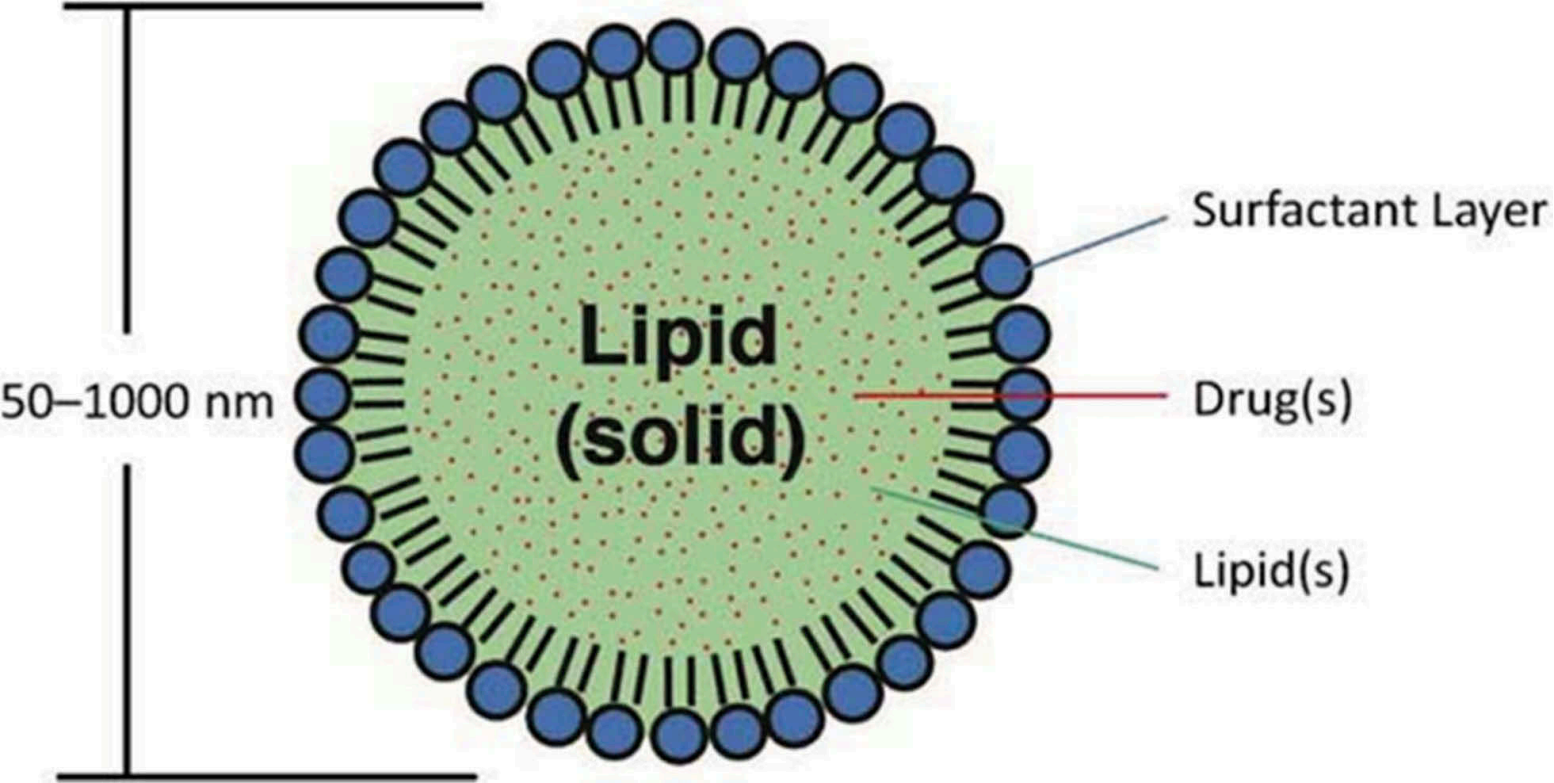
Schematic diagram of solid lipid nanoparticles [99].

Solid lipid nanoparticles (SLNs) shown in Figure 5 have also been tested. They are generated by exchanging the liquid lipid of emulsions for a solid lipid, making them solid at room and body temperature, thus inducing a reduction in particle size. The capacity of SLN:DNA vectors to induce the expression of a foreign protein after intravenous administration has been demonstrated. By neutralizing the acidic pH inside the endosome, DNA molecules, protected against acid attack of protons, remain intact inside the cell. The most important difference with liposomes is that the core matrix used for release and delivery of bioactive substances becomes lipophilic instead of aqueous for liposomes [98]. Some advantages of SLNs are their low toxicity, good storage stability, and the possibility of steam sterilization and lyophilization.

#### 4.2.3. Cationic cholesterol disulfide lipids

The use of cholesterol and some of its derivatives for the synthesis of gene delivery carriers has been recently tested. Cationic cholesterol disulfide lipids (CHOSS) were synthesized by binding cholesterol to cationic head groups via a disulfide linkage. Headgroups which were used included histidine, pyrimidine, or methyl imidazole, which demonstrated low cytotoxicity and high transfection efficiency. CHOSS can bind to DNA with high affinity forming stable lipoplex NPs. Transfection experiments with COS-7 cells confirmed the low cytotoxicity and particularly high uptake capability of these particles [100]. Interestingly, the particles were specifically localized to the periphery of cell nuclei following uptake. Once inside the cell, the release of the DNA may occur via cleavage of the disulfide bond in the reducing intracellular environment.

#### 4.2.4. Biomimetic engineering

Another approach has been created which is known as biomimetic nanocapsules. They consist of an oily core made up of a mixture of triglycerides and polyglyceryl-6 dioleate surrounded by a shell of free polyethylene glycol (PEG) and HS-PEG. They have been used for DNA encapsulation to improve the resistance to rapid clearance. Through PEG coating, the positive charges of such complexes are masked and thus prevent opsonization by immune proteins. Furthermore, because PEG leads to destabilization of the lipid endosomal membrane, DNA nanocapsules are better than lipoplexes when the limiting step in the transfection of endosomes has to be avoided [101]. Another important consideration is the biodegradability of the nanospheres once they have traversed the required specific sites. Early biopolymer NP design focused primarily on the use of nonbiodegradable synthetic polymers such as polyacrylamide and poly(methylacrylate). The risks of chronic toxicity due to the intracellular and/or tissue overloading of nondegradable polymers were soon considered as a major limitation for the systemic administration of polyacrylamide and poly(methylacrylate) NPs in humans. As a consequence, the focus has shifted toward NPs designed using synthetic biodegradable polymers including polyalkylcyanoacrylate, poly(lactic-co-glycolic acid), and polyanhydride [102]. The therapeutic potential of these biodegradable colloidal systems was investigated for various applications.

There is another limitation for the bionanoparticle-based administration of hydrophilic molecules including nucleic acids (oligonucleotides and genes) [103]. This limitation is mainly because the polymers forming these NPs are mostly hydrophobic, while biomolecules including nucleic acids are hydrophilic. This leads to difficulties of efficient encapsulation and protection against enzymatic degradation. Therefore, the preparation of NPs using more hydrophilic and naturally occurring materials has been explored.

#### 4.2.5. Dendrimer-based DNA engineering

**Figure 6.**
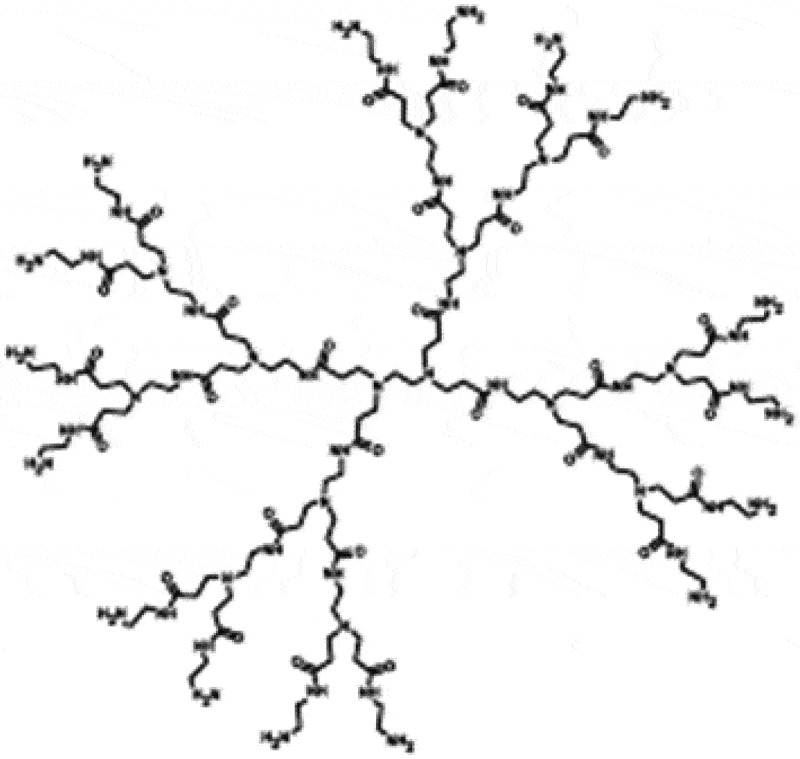
PAMAM dendrimers [106].

Dendrimers have attracted interest for drug and gene delivery systems because they possess a number of unique and interesting characteristics such as defined structures, inner cavities able to encapsulate guest molecules, and controllable multivalent functionalities in their inner or outer parts [104]. Figure 6 shows the repetitively branched molecules of dendrimers composed of poly(amido amine) monomers. These properties make dendrimers an important option for the development of nanoscale nonviral vectors for nucleic acid delivery. Dendrimers can interact with various forms of nucleic acids (i.e. DNA, RNA, and oligonucleotides). These interactions, primarily electrostatic, lead to complexes, which protect the nucleic acid from degradation. The properties of these complexes depend on many factors, such as stoichiometry, the concentration of dendrimer amines and nucleic acid phosphates, solvent properties like pH, and salt concentration [105]. However, the inherent difficulty of synthesizing new dendrimers that are suitable carriers for drug delivery has led researchers to focus primarily on the modification of existing dendrimers, instead of the development of novel dendrimers for gene delivery systems.

Poly(amido amine) (PAMAM) dendrimers have been tested as genetic material carriers and have been also modified with PEG, amino acids, or ligands in order to enhance their gene delivery potency. Numerous reports have been published describing the use of amino-terminated PAMAM or PPI (poly(propileneimine)) dendrimers as nonviral gene transfer agents, enhancing the transfection of DNA by endocytosis and, ultimately, into the cell nucleus [107]. The observed high transfection efficiency of dendrimers may not only be due to their well-defined structure but may also be caused by the low pKa of the amines, which permits the dendrimer to buffer the pH change in the endosomal compartment. Dendrimers hold a promising future for various biomedical applications for the coming years, as they possess unique properties, such as a high degree of branching and multivalency. Also, as research progresses, newer applications of dendrimers will emerge, and the future should witness an increasing number of commercialized dendrimer-based DNA delivery systems.

#### 4.2.6. Protein-based engineered colloidal

The first naturally occurring material used for the preparation of NPs consisted of two proteins, albumin and gelatin. Protein-based colloidal systems are very promising because they are biodegradable, nontoxic, and less immunogenic. They have greater stability in vivo and during storage are relatively easy to prepare and to monitor size distribution [108]. In addition, because of the defined primary structure of proteins, protein-based NPs offer various possibilities for surface modification and covalent drug attachment. For all these reasons, a number of proteins have been used to develop protein-based NPs for drug delivery. These include albumin, collagen, gelatin, fibroin, sericin, and keratin. As an example, gelatin is one of the most versatile natural biopolymers derived from collagen, and it has been widely used in food products and medicines. Many researchers have used gelatin NPs as a gene delivery vehicle. With solvent displacement, type B gelatin, derived from alkaline hydrolysis of collagen, which has an isoelectric point at around, can physically encapsulate nucleic acid constructs at neutral pH. Furthermore, the physical encapsulation in gelatin NPs preserves the supercoiled structure of the plasmid DNA and improves the transfection efficiency upon intracellular delivery [109].

An approach which has recently been used for gene delivery includes genetically engineered protein-based polymers, which incorporate peptide motifs such as elastin, silk, and collagen. This approach has the advantage that the properties of the resulting NPs can be tailored to avoid cytotoxicity and rapid clearance, while ensuring delivery of the DNA package to the intended target [110]. Another promising approach, which has primarily been tested in vitro, combines the properties of lipids and peptides to achieve high efficiency of transfection with minimal toxicity even in the presence of serum. This approach consists of the use of oligoarginine–lipid conjugates. The lipidic part of these complexes consists of 3,5-bis(dodecyloxy)benzamide (BDB) and a PEG spacer which is introduced between the amide group of BDB and the C-terminal of oligo-Arg.

Polysaccharide-derived NP surfaces help to improve the biocompatibility of cell toxic material, together with new immobilization approaches, which are currently in development for novel bionanoparticle-derived pharmaceutical formulations [111]. NPs from naturally occurring polysaccharides were designed for the administration of peptides and proteins, as well as nucleic acids. An important example of this approach is the use of chitosan, a natural biodegradable cationic polysaccharide derived from chitin consisting of D-glucosamine and N-acetyl-D-glucosamine. It is produced by deacetylation of chitin extracted from the shells of crabs, shrimp, and krill. This linear polymer has been shown to be biocompatible and nonimmunogenic and to possess mucoadhesive properties, making it an excellent biopolymer for the preparation of NPs as vectors for DNA delivery [112]. Indeed, chitosan can spontaneously bind to DNA via ionic interactions to form NPs. The biochemical properties of chitosan make it a suitable vehicle for gene transfection. The amino groups confer to the molecule a high charge density and are readily available for chemical modification and salt formation with acids. In vivo, it is degraded by lysozyme and it has been also shown to partially protect DNA from nuclease degradation [113]. Cellular transfection with chitosan–DNA complexes has demonstrated that once inside the cell, complexes of 100–250 nm can be found accumulated in the nucleus. Finally, chitosan–DNA nanospheres have been shown to be nontoxic in both experimental animals and humans.

### 4.3. Environmental interactions

Interactions with serum components can be minimized by modifying the surface of polymeric NPs with hydrophilic polymer chains in a process called passive targeting as it allows the modified NPs to avoid the body’s clearance mechanisms, increasing the chances that they will reach their intended target. There are several ways to achieve passive targeting such as surface modification of polymeric NPs with hydrophilic polymer chains or incorporation of environmentally insensitive polymers into the nanoparticles. A dense, hydrophilic shell of long chains is formed that protect the core from interacting with solutes, including nonspecific hydrophobic interactions with the reticuloendothelial system. This polymeric protection is referred to as ‘steric stabilization'. Modifications of the cationic polymers, such as PEGylation, have been incorporated to facilitate delivery and enhance transgene expression. At the same time, the terminal hydroxyl groups of PEGcan be derivatized, leading to monofunctional, homo-, or hetero-bifunctional and even multi-arm PEG, allowing further conjugation of selected ligands.

The type B gelatin-based NPs has been used as a noncondensing gene delivery system for tumor targeting [114]. They prepared unmodified and PEG-modified gelatin NPs by ethanol precipitation, leading to particles in the range of 200–500 nm. Tetramethylrhodamine-dextran, a hydrophilic fluorescently labeled molecule, was first used as model drug for in vitro cell uptake studies. The control and PEG-modified type B gelatin NPs were taken up by cells through nonspecific endocytosis [115]. Within 12 h of PEG-modified gelatin NP internalization by NIH-3T3 murine fibroblasts, the payload was released and accumulated around the perinuclear region. Further experiments using encapsulated plasmid DNA, encoding for enhanced green fluorescence protein (EGFP-N1), confirmed the long-lasting transgene expression potential of PEG-modified type B gelatin NPs compared to other methods. In addition, neither gelatin nor PEG-modified gelatin NPs demonstrated any toxicity. To complement the in vitro evaluations, the biodistribution profiles of unmodified and PEG-modified ^125^Iodine (^125^I)-labeled gelatin NPs following intravenous administration through the tail vein in Lewis lung carcinoma-bearing C57BL/6 J mice were further examined [116]. PEG-modified NPs remained in circulation for an extended period and preferentially accumulated in the tumor for up to 24 h postadministration as well as the liver. Conversely, unmodified NPs were rapidly cleared from the circulation and remained mostly in the liver and spleen. These results show that PEG-modified gelatin NPs can be passively targeted to the tumor mass following systemic administration and have the potential to be an effective vector for anticancer gene therapy. In another study, the potential of PEG-modified gelatin NPs for passively targeting gene delivery to tumors were further demonstrated. Interestingly, intravenous administration of an encapsulated reporter plasmid DNA into tumor-bearing mice eventually led to a higher level of transfection of the tumor than following intratumoral administration [117]. A more active tumor-targeting strategy takes advantage of the fact that tumors express elevated levels of glutathione (GSH) compared to normal cells [118]. GSH is a tripeptide, generally expressed in the cell cytoplasm and functions as an antioxidant to prevent damage related to the reactive oxygen species. The intracellular GSH concentration (5–10 mM) is generally higher than the extracellular concentrations (1–10 mM). During active proliferation of tumor cells, GSH and peroxide levels are further elevated in the cytoplasm. Introduction of thiol groups is a common modification that can allow for intracellular delivery through the reduction of disulfide cross-links [119]. This approach has been tested in vitro and leads to increased transfection efficiency compared to other NP-based systems.

Chitosan–DNA nanospheres have also been tested for clinical applications. Several delivery routes of chitosan–DNA NPs have been investigated in animal models including intranasal and oral delivery, resulting in transduced gene expression in the stomach and intestinal epithelium of a peanut allergen gene providing protection against allergen-induced anaphylaxis [120]. In addition, such NPs have also been designed for tissue engineering and local gene delivery in periodontal tissue regeneration.

### 4.4. Intracellular trafficking

Following extracellular transport to the target cell, the vector is internalized primarily by endocytosis and then must escape the endosomal compartment to avoid lysosomal degradation before transport to the nucleus and crossing the nuclear membrane for subsequent transcription [121]. Nuclear localization occurs either following cell division or through transport through the nuclear pore complex. The rate-limiting step for this process is dependent upon the vector. Lipoplexes exhibit poor cellular uptake, whereas polyplexes exhibit limited nuclear import. Small-molecule peptides and proteins such as transferrin, antibodies, or galactose (for hepatocyte targeting) have been attached to either the plasmid or to the cationic lipid or polymer to promote endosomal escape and nuclear localization [122].

Fusogenic peptides have been incorporated to disrupt membranes, which may facilitate either cell entry without endocytosis or endosomal escape following endocytosis. Nuclear targeting can be enhanced by the attachment of nuclear localization signals (NLS), which are oligopeptides that bind to importins, cytoplasmic receptors responsible for binding and transport through the nuclear pore complex. For example, NLS addition to the polysaccharide chitosan improved transfection efficiency. However, success with NLS has been varied: while some papers have reported that inclusion of NLS-containing proteins or peptides increases gene transfer and expression, such as for chitosan, others have found no such enhancement [123].

Numerous opportunities remain to increase gene transfer by virus mimicking: that is the design of vectors to interface with specific cellular processes, with functional groups derived from the understanding viruses or cellular processes. Nonviral vectors engineered with functional groups that allow for directed motion along the cytoskeleton could increase accumulation at the nucleus and decrease the amount of DNA required [124]. Peptide nucleic acids (PNAs) have been developed that are able to bind tightly to specific DNA sequences. Incorporation of PNAs could potentially target the transgene to a specific chromosomal location as some viruses do. Alternatively, engineered zinc-finger proteins with nuclease activity were developed to recognize a unique chromosomal site and induce a double-strand break [125]. At this break site, the chromosome can recombine with an extrachromosomal sequence of interest. Finally, the nucleic acid itself could be engineered to avoid silencing by methylation, thereby extending the duration of transgene expression.

### 4.5. Immunoassays

Immunoassays play a vital role in laboratory research, clinical diagnostics, and food and environmental monitoring. They combine immunology and chemistry to create scientific tests such as enzyme immunoassays for the specific and sensitive detection of the analytes of interest. These assays are based on the principle of the specificity of the interaction between the antibody and its cognate antigen. Immunoassays are relatively easy to perform contributing to their widespread use. Radioimmuneassays (RIAs) and enzyme immunoassays such as ELISA (enzyme-linked immunosorbent assay), luminescent immunoassays, and fluorescent immunoassays (FIAs) are all currently used. The antibodies can be labeled in several ways including radioisotopes, fluorescent dyes, or enzymes that catalyze fluorogenic or luminogenic reactions, thus allowing visualization of the antibody–antigen interaction [126].

In the past, RIAs were widely used but have been slowly replaced by assays using fluorescent molecules and enzymes as labels to avoid the obvious disadvantages of radioisotopes. Nevertheless, the use of enzymes and fluorescent labels is often less straightforward and do not yet achieve the sensitivity and limits of detection of radioisotope-based assays. When first developed in the 1970 s, ELISAs and Western blot assays provided a significant increase in sensitivity over existing detection methods [127]. Today, a wide variety of enzyme-linked antibodies with choices of chemiluminescent, bioluminescent, chemifluorescent, fluorescent, and more traditional colorimetric detection systems are commercially available from various suppliers. Despite recent advances, there is an urgent need to improve immunoassay sensitivity for multiple applications such as the deciphering of normal and pathophysiological biological processes, early disease detection, or the detection of environmental hazards, all instances where marker levels may be very low.

Antibody–enzyme (Enz-Ab) conjugates are most often prepared by crosslinking enzymes to the antibody via their functional groups such as the primary amines and sulfhydryls, or by cross-linking through sugar moieties attached to one of the proteins. Chemical activation of the target residues on both the enzyme and the antibody tends to be random and difficult to control. Thus, the use of homobifunctional reagents such as glutaraldehyde often results in very low yields, and the resulting Enz-Ab complexes can be heterogeneous and highly polymerized [128]. Such Enz-Ab complexes can compromise the sensitivity of the subsequent immunological assay due to steric hindrance, which affects antigen-binding capacity. In addition, large-molecular-weight oligomers formed by uncontrolled cross-linking may produce insoluble aggregates that are impossible to manipulate in immunoassays. In the case of glycoproteins, some of these difficulties can be circumvented by periodate oxidation. This method has been used to cross-link horseradish peroxidase (HRP), a glycoprotein, to functional groups on an antibody in a more controlled way [129]. However, this approach is limited to enzymes that contain carbohydrate moieties and thus is not applicable to all enzymes including the commonly used reporter enzyme alkaline phosphatase.

In FIAs, signal amplification is typically achieved by coupling fluorophores, such as organic dyes, to the antibody probes. The sensitivity of FIAs is mainly determined by the number of light quanta emitted/analyte molecule. Increasing the fluorescent dye to Ab ratio results in improved signal amplification and therefore sensitivity [130]. As for Enz-Ab conjugates, labeling antibodies with large numbers of fluorophores usually leads to reduced specificity and binding affinity as well as a reduced quantum yield due to dye self-quenching effects. For these reasons, the F/P ratio is normally kept around 4–8, thus limiting the sensitivity of the assays.

### 4.6. Functionalization of nanoparticles by biological entities

The creation of NPs with the desired physiobiochemical properties remains a challenge. First, it is important that the antibody molecules are stably attached to the particle surfaces while maintaining their ability to interact with the target analyte. In addition, NP aggregation and their nonspecific binding with biological molecules remain a serious issue. For example, proteins can be either hydrophilic or hydrophobic, with either negative or positive charges, making it very difficult to avoid nonspecific interactions. To address these technical challenges, numerous surface modifications and immobilization procedures have been explored and developed [131].

A common surface modification strategy is postcoating and modification of the NP surface with different functional groups, including carboxylate, amine, PEG, or combinations of different functionalities. Alternatively, the direct synthesis on the surface of the NPs of a mixed monolayer of PEG and a functional group has been tested. While the ethylene glycol chains, which are water soluble and neutral in charge, function as a shielding component to minimize nonspecific binding, the functional group can be used as a capture agent for antibody conjugation acts. Immobilization of biomolecules on surfaces can be achieved in a number of ways via adsorption, by chemical linkage, or by affinity-based interactions.

In adsorption, molecules are adsorbed at the interface via physical forces such as van der Waals, electrostatic, or hydrophobic interactions depending on the chemical nature of the surfaces and molecules. Consequently, the conditions used for immobilization are highly sensitive to ionic strength, pH, and temperature, leading under certain conditions to subsequent dissociation of biomolecules [132]. Moreover, this method of immobilization may interfere with proper folding and can lead to multilayer adsorption and loss of orientation or enzymatic activity. However, because adsorption of biomolecular targets is relatively simple to perform, it is widely used. The disadvantages of adsorption can be overcome to some extent by covalently linking biomolecules to the particle surface via amide, ester, ether, or sulfide bonds. Because of the large variety of reactive groups found on organic NPs made of proteins, polysaccharides, lipids, or polymers, the well-defined methods of chemical modification are often not position specific and therefore lack orientation [133]. In some cases, the reactive groups are located close to the active center, affecting activity and function.

Streptavidin–biotin interactions are widely used for biotechnology applications, in particular immunoassays. Although biotin–streptavidin binding is not covalent, its high-affinity constant produces a highly specific and nearly irreversible immobilization. The streptavidin–biotin interaction is the strongest noncovalent receptor–ligand interaction (K_a_ = 10^15^ M^−^1) currently known, with a stronger affinity than in any known antigen–antibody interaction [134]. Bond formation between biotin and streptavidin is very rapid, and once formed, it is unaffected by most extremes of pH, temperature, organic solvents, and other denaturing agents. Streptavidin contains four subunits, each with a single biotin-binding site, allowing signal amplification.

A variety of biomolecules can be biotinylated and subsequently used with streptavidin labeled probes [135]. Biotinylated antibodies, which serve as recognition agents, are used in a variety of different assays, and therefore, many conjugates are now commercially available. As such, streptavidin linkage is a very convenient and efficient conjugation method to attach recognition agents onto NPs. An easy way of immobilizing streptavidin onto a surface is based on electrostatic interaction. The positively charged streptavidin naturally adsorbs to a negatively charged surface and can then be stabilized by cross-linking using a bifunctional reagent such as glutaraldehyde [136]. Resulting streptavidin-coated particles can then be functionalized by conjugation to biotinylated recognition molecules.

An interesting alternative to the streptavidin–biotin system called ‘protein-assisted nanoassembler’ has been recently described. This approach allows the robust self-assembly of multifunctional superstructures consisting of different single-function particles such as labels, carriers, recognition, and targeting agents, including antibodies. This bioengineering method employs two unique proteins from *Bacillus amyloliquefaciens, barnase* and *barstar*, to rapidly bring together the structural components directly in solution [137]. The properties of the superstructures can be designed on demand by linking different agents of various sizes and chemical nature, as a function of the specific purpose. It has been demonstrated that using barnase and barstar, it is possible to assemble colloidally stable trifunctional structures by binding together magnetic particles, QDs, and antibodies. Indeed, the bonds between these proteins are strong enough to hold macroscopic (5 nm–3 mm) particles together. Specific interaction of such superstructures with cancer cells resulted in fluorescent labeling of the cells and their responsiveness to a magnetic field. This very recent and versatile method can be used for multiple nanotechnology applications including immunoassays.

### 4.7. Bioanalytical applications

Numerous efforts have been made to optimize antibody labeling in order to further improve the performance of immunoassays. In a careful study, the optimization of biotin labeling of a mouse IgG was done by varying the classical parameters of the labeling protocol [138]. The immobilization of biotin-tagged mouse IgGs on avidin-coated plates was then investigated by incubating the bound antibodies with goat anti-mouse IgGs linked to fluorescent beads. The optimum conditions were successfully applied in sandwich immunoassays for two different analytes, resulting in the detection of as little as 2 and 5 ng of troponin I and N-terminal probrain natriuretic peptide (BNP), respectively. In an interesting but still little used approach, Simons et al. described the covalent in vacuo cross-linking of HRP to anti-rabbit immunoglobulin G (IgG). The advantageous feature of this co-lyophilization-based procedure is that the cross-linking to form Enz-Ab conjugates is accomplished without the use of chemical modifying or activating reagents, reducing the potential activity loss due to chemical modification. The resulting soluble multienzyme–IgG immunoconjugate exhibited a 100-fold increased sensitivity for antigen detection compared to a commercial conjugate prepared by conventional chemical cross-linking methods.

The uses of NPs provide an interesting and powerful avenue to achieve significant signal amplification by allowing the linkage of a single antibody molecule with up to thousands of reporter molecules such as fluorophores or enzymes [139]. In an interesting example of this strategy, a polypeptide containing 20 lysine residues each conjugated to an HRP molecule was attached to 0.44 mm streptavidin polystyrene spherical NPs introducing hundreds of HRP molecules and making a signal amplifying detector conjugate. Such highly labeled NPs were further functionalized with IgG molecules to achieve a molar ratio of 1 IgG to 105 HRP complexes. These immunoconjugates efficiently bound to plasma anti-HIV-1 antibodies that had been captured by HIV antigens on 5 mm carboxyl magnetic microparticles and produced a detection signal with five to eight times more sensitivity as compared to conventional HRP conjugated goat anti-human IgG [140].

Similarly, sulfate functionalized polystyrene NPs were used to electrostatically attract positively charged antibodies. Protein immobilization maintained the Y-shaped orientation of the molecules as well as their immunological activity, optimizing the sensitivity of the immunosensor. Such complexes tested in sandwich immunoassays conducted to detect cardiac troponin I showed a fivefold higher activity over the control [141].

The sensitivity of these assays is generally limited by the ratio of label (fluorescent or enzyme) molecules per biomolecule (L/P ratio). The L/P ratio is typically 4–8 for a conventional, covalently coupled fluorescent immunolabel, for example, an IgG labeled with fluorescein isothiocyanate (IgG-FITC) conjugate. A higher L/P may lead to a decrease of the specific binding affinity of the biomolecule, and additionally cause self-quenching effects [142]. Increasing the effective dye/biomolecule ratio while minimizing dye self-quenching and maintaining the biomolecule’s binding properties is thus an important goal in assay development. Several ways to increase the F/P ratio have been investigated, in particular those which are based on coprecipitation or self-assembly without the formation of covalent bonds. One approach has been the substitution of labeling molecules by micro- or nanocrystalline dyes. For example, perylene, a fluorescent polycyclic aromatic hydrocarbon consisting of two molecules of naphthalene that have been fused together, has been used as label. A higher F/P ratio can be obtained by precipitating fluorescent perylene microparticles in the presence of the antibodies. After the immunoreaction, a large number of fluorescent molecules contained in these particles can then be dissolved in a suitable solvent for detection. An analogous route was to link antibodies to polyelectrolyte encapsulated microcrystalline fluorescent material [143]. The surface of these particulate structures is typically engineered by the layer-wise assembly of oppositely charged polyelectrolytes, the outer layer consisting of biorecognition molecules, for example, immunoglobulins. Because of the exceptionally high F/P ratio of the detection antibodies, a dramatically amplified immunoassay was achieved. Despite the advantages of these two methods, a key limitation lies in the restricted number of materials that can be precipitated or crystallized for encapsulation. Given the limitations of existing fluorescence-based biochemical assays, the development of new strategies and biolabeling systems will be necessary.

**Figure 7.**
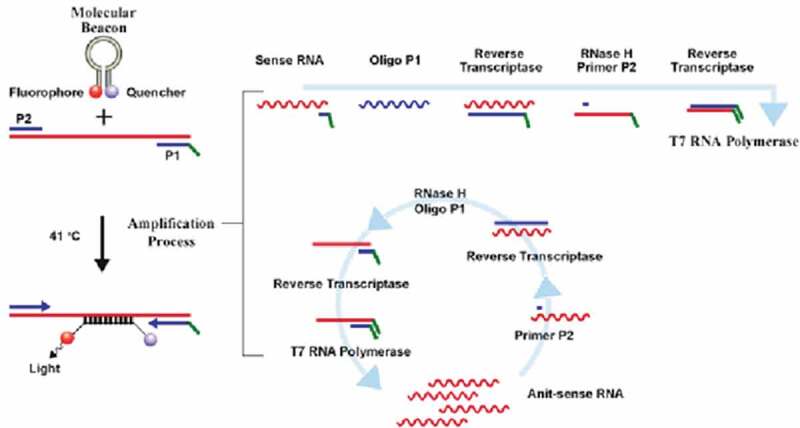
Overall process of amplification technology [145].

A novel signal amplification technology based on a new class of biofunctional fluorescent nanocrystals has been developed, consisting of a two-step approach to encapsulate the fluorogenic precursor fluorescein diacetate (FDA) nanocrystals followed by conjugation of the antibody [144]. Figure 7 shows the overall process of amplification technology. Distearoylphosphatidylethanolamine modified with PEG (2000) amine is coated on the surface of the FDA nanocrystals to provide an interface for antibody coupling. Anti-mouse antibodies are subsequently attached to the nanocrystalline FDA biolabels by adsorption.

A high molar ratio of fluorescent molecules to biomolecules is achieved in this nanocrystal biolabel system. The analytical performance of the nanocrystal-based label system has been evaluated in a model sandwich immunoassay for the detection of mouse IgG. The high sensitivity of this assay may be explained by the boosting effect of the high ratio of dye/antibody, but washing out the dye molecules after the affinity reaction may also contribute to improve the signal, the mean dye-to-dye distances being large enough to diminish the quenching effects.

Other high-load systems have been successfully designed and used as immunolabels such as fluorescent conjugated dendrimers, fluorophore-loaded latex beads, and liposome-encapsulated fluorophores. For example, liposomes used in immunodetection and drug delivery systems can be tagged with antibodies to form immunoliposomes. Numerous procedures for the conjugation of antibodies to liposomes have been developed, falling into four general categories defined by the particular functionality of the antibody being modified, namely amine modification, carbohydrate modification, disulfide modification, and noncovalent conjugation. Interestingly, studies showed inserted a monosialoganglioside (GM1), which exhibits a specific affinity toward cholera toxin (CT), into the phospholipid bilayer during the liposome synthesis [146]. These GM1-sensitized, sulforhodamine B dye-entrapping liposomes were then used for the determination of CT. Subsequently, this group reported the successful preparation of biotin-tagged, carboxyfluorescein-encapsulated liposomes by using the reversed-phase evaporation method from a lipid mixture containing biotin-X-DHPE. Such liposomes have been successfully used to improve biosensing systems. Carboxy-encapsulated fluorescein biotin-tagged liposomes were used as a novel alternative analytical method for the detection of low concentration of biotin.

### 4.8. Nanoparticles and the fundamental study of cell adhesion mechanisms

Adhesion is a highly important and fundamental phenomenon in biology. Living cells are endowed with different receptors expressed at the plasma membrane that allow the continuous perception of the extracellular environment. These ubiquitously present receptors are quite diverse in function and include, in particular, receptors that anchor cells to the extracellular matrix (integrins) or those involved in cell–cell interactions (such as selectins or cadherins).

The study of cell adhesion has to be tackled using multiple approaches, from molecular, developmental, or cell biology to biophysics [147]. The field of the study of cell adhesion is thus, by nature, multidisciplinary, involving a large number of research groups producing an ever-increasing number of publications as our understanding of this complex process grows.

Intercellular contacts, created by morphologically distinct structures, are made of the clustering of cell-surface transmembrane adhesive receptors into multiprotein assemblies, or junctions, connected to the cytoskeleton and intracellular signaling pathways [148]. Numerous molecules are involved which regulate various mechanisms such as differentiation, migration, or apoptosis.

Adherens junctions consist of complex intercellular structures formed by localized clusters of trans dimers between classical cadherins from apposed cells. Cadherins are single-pass transmembrane glycoproteins and signal transducing molecules involved in Ca2þ-dependent homophilic cell–cell adhesion. During development, cadherins contribute to the regulation of a large number of processes, including tissue morphogenesis such as mesenchymal–epithelial transition, cell sorting and tissue rearrangement through convergence extension, neurite elongation, and synaptogenesis. In addition, deregulation of cadherin-mediated adhesion has been associated with alterations of tissue homeostasis. Thus, cadherins are key morphoregulatory molecules in developmental processes, as well as essential contributors to cell–cell cohesion within adult tissues and organs [149]. A comprehensive view of cadherin recruitment and dynamics at cell–cell contacts and its regulation is of major importance for the understanding of the control of cell fate in normal as well as pathological situations, such as carcinogenesis.

Classical cadherins (type I and type II) consist of an extracellular segment typically containing five tandem repeats of an approximately 110-amino acid module, numbered EC1 to EC5 from the outermost domain, a transmembrane region, and a highly conserved cytoplasmic domain. Cadherin engagement triggers a series of still only partially understood intracellular signaling events that lead to the reorganization of the actin cytoskeleton via cytoplasmic proteins such as catenins, plakoglobin, and p-120 that, in turn, trigger changes in cell morphology and motility [150]. The formation of adherens junctions is likely to represent the first step in this signaling cascade.

Despite detailed studies of cadherin-mediated adhesion in multicellular organisms, the molecular understanding of the adhesive states of cadherin is less clear. Cadherin expression is cell type or tissue specific, and a cell type may express more than one type of cadherin. Cells expressing cadherins sort out and aggregate only with cells expressing identical cadherins. This is the basis of tissue patterning and architecture in both cell-to-cell contact and cell migration. How these molecules interact with each other and the mechanisms by which they transfer specific intracellular signals remain poorly understood.

Structural studies have shown that cadherin–cadherin contacts are mediated by the cadherin extracellular domain. A detailed structural description of adherens junctions is emerging from the elucidation of the structure of individual molecular partners. However, despite numerous studies over the past 25 years, the details of trans dimerization are still under debate. The crystal structures of the entire (EC15) ectodomain from classical type I C-cadherin and more recently E- and N-cadherins reveal a ‘strand swap’ trans interface in which the N-terminal b-strand from the EC1 domain of each paired cadherin exchanges with that of the partner molecule. A second functionally important trans interface, involving the linker region between the EC1 and EC2 domains, has also been identified and constitutes a kinetic intermediate on the path to the formation of strand swapped dimers. However, a widely accepted model as summarized in recent review articles is that the functional unit of cadherin adhesion is a cis dimer formed by binding of the extracellular domains of two cadherins on the same cell surface [151]. The interplay between trans binding and lateral cis interactions among proteins on the same membrane theoretically plays a crucial role in the clustering of cadherins into junctions, but evidence for these trans- and cis-cadherin-binding states remains controversial.

### 4.9. Polymeric beads

#### 4.9.1. Bead–bead and bead–cell assays

**Figure 8.**
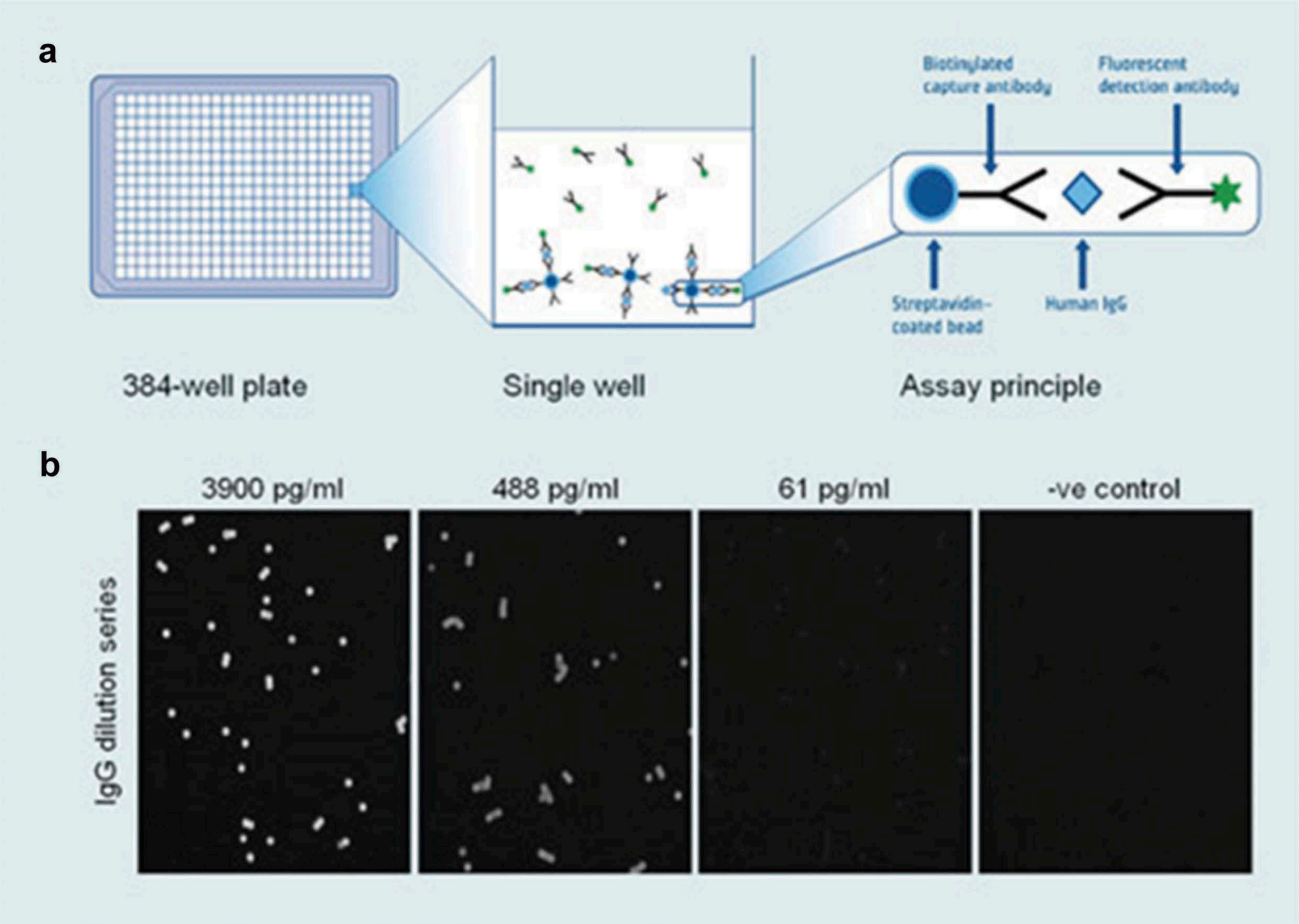
Bead-based assay [152].

Cell aggregation assays were often used in early studies to evaluate the role of cadherins on cell–cell adhesion. Such studies are challenging in live cells, and this approach provides mostly qualitative data as it is difficult to take into account the number of cadherin molecules involved. Moreover, cadherins are multimodular and involved in complex multimolecular structures, making it difficult to elucidate their biological properties. For this reason, glass and polymeric NPs have been often used in standard assays to assess the biological activity of cadherin recombinant fragments. Fluorescent protein A-coated 0.9 mM polystyrene beads functionalized with chimeric C-cadherin fragments and streptavidin-coated 2.8 mm polystyrene beads decorated with E-cadherin fragments were used to address the involvement of the different ectodomain modules in bead aggregation assays. Figure 8 reveals the bead-based assay principles with regard to the cell aggregation.

Bead–cell-binding assays have also been used to dissect the molecular mechanisms of signal transduction [153]. N (neural)-cadherin chimera-loaded latex beads of 6-mm diameter self-aggregate and specifically bind, in an aCa2þ-dependent manner to N-cadherin-expressing cells. N-cadherin full-length chimera-coated beads fully mimic cadherin-mediated cell–cell interactions, inducing the accumulation of N-cadherin, catenins, and F-actin as well as membrane remodeling at the bead–cell contact. Streptavidin spherical polystyrene beads (2.8 mm in diameter) decorated with E/EC12 have been shown to efficiently interact with HC11 cells and activate membrane dynamics events. Indeed, subsequent to these contacts, the beads were rapidly engulfed by the E-cadherin-expressing cells, and this internalization appears to be highly specific and sensitive to point mutations [154].

#### 4.9.2. Single-molecule assays

Most approaches used to study cadherin–cadherin interactions provide information from the behavior of multimolecular systems with an often incompletely defined geometrical organization. Many contradictory results and unanswered questions suggest that it would be a hopeless task to derive clear molecular properties from these data. An understanding of the intrinsic kinetic properties of cadherin interactions requires the measurement of parameters at the single-molecule level. During the past 15 years, new biophysical approaches have been developed that allow the study of ligand–receptor interactions with unprecedented accuracy, down to the single bond level [155]. Reported results include information on bond mechanical properties, association behaviors of surface-attached molecules, and the dissection of energy landscapes and reaction pathways. Indeed, monitoring single bond formation and dissociation has made it possible to bypass difficult problems such as force sharing between multiple bonds or assessing the effect of partial geometrical matches on the kinetics of bond formation [156]. Several of these methodologies have been used to study cadherin interactions at the molecular level. A surface force apparatus has been used to investigate the mechanisms of cadherin binding by measuring the force/distance between cadherin ectodomains. Single-molecule atomic force microscopy (AFM) was used to study the mechanical resistance of cadherin interactions. However, interpretation of the data was not straightforward: the relationship between the unbinding force, as measured by AFM, and the dissociation rate is complex and dependent on the cantilever stiffness and rate of sample displacement [157]. At the present time, these experiments have yet to give us a comprehensive view of the mechanisms underlying cadherin interactions.

Two approaches, the flow chamber and the Biomembrane Force Probe (BFP), offer a way to bring into contact two surfaces covered with molecules and a means of measuring the duration of the interaction as well as the rupture force under stress. The molecules to be tested are linked in the case of the BFP approach onto two micron-sized glass beads, whereas in the flow chamber approach, they are linked onto a polystyrene bead and a flat surface [158]. These methodologies allow the analysis of the association of surface-attached rather than soluble molecules reproducing a molecular orientation relevant to physiological conditions, highly sensitive detection of molecular interactions, and the kinetic study of bond formation and dissociation, under stress. These two approaches provided the first quantitative data describing the dissociation kinetics of individual E-cadherin and C-cadherin interactions.

## 5. Conclusion and perspective

Nanomaterials, notable for their extremely small feature size, have the potential for wide-ranging industrial, biomedical, and electronic applications. They are excellent adsorbents, catalysts, and sensors due to their large specific surface area and high reactivity. NPs play an important role in many biotechnology applications and promise to take center stage for many new and emerging applications in the coming years. Interesting future developments include not only biomedical applications such as improved delivery of drugs to tumor cells and the use of dendrimers for regenerative medicine but also fields such as water purification and disinfection, food production, and packaging. It will be attractive to find a simple, efficient, and controllable way to produce nanomaterials in mass production as well as to bridge their applications in optoelectronics.
